# *Neisseria meningitidis* Serogroup X Sequence Type 2888, Italy

**DOI:** 10.3201/eid1602.091553

**Published:** 2010-02

**Authors:** Cecilia Fazio, Stefania Starnino, Marina Dal Soldà, Tonino Sofia, Arianna Neri, Paola Mastrantonio, Paola Stefanelli

**Affiliations:** Istituto Superiore di Sanità, Rome, Italy (C. Fazio, S. Starnino, T. Sofia, A. Neri, P. Mastrantonio, P. Stefanelli); Azienda Sanitaria, Locale, Cremona, Italy (M. Dal Soldá)

**Keywords:** Neisseria meningitidis, meningococcal infections, serogroup, genotype, bacteria, Italy, letter

**To the Editor:** *Neisseria meningitidis* serogroup X was first described in the 1960s and has been found to be responsible of rare cases of invasive meningococcal diseases, in particular, meningitis, in North America, Europe, Australia, Africa, and the People’s Republic of China (*1*–*3*). This serogroup has recently emerged in Africa as an increasing cause of meningitis; unfortunately, it is not covered by current vaccine programs. Serogroup X outbreaks have been reported in Niger, Ghana, and Kenya (*4*–*6*). In particular, in Niger during January–June 2006, *N. meningitidis* serogroup X represented 51% of confirmed cases of meningitis (*4*).

To investigate the population structure of serogroup X meningococci isolated during recent decades in Africa, Europe, and North America, Gagneux et al. (*1*) compared the molecular characteristics among them. That study highlighted a low genetic variability between African serogroup X strains, which contrasts with higher genetic variability among isolates from Europe and the United States (*1*).

We describe a case of invasive meningococcal disease caused by a serogroup X *N. meningitidis* strain isolated in Italy. The patient was a 55-year-old Italian woman with no immune deficiency. The onset of disease started quickly with high fever (39°C) on June 1, 2009. No contacts with persons coming from abroad were reported. This case was diagnosed on the basis of clinical signs and symptoms and results of laboratory confirmatory tests, including blood culture. The patient received ceftriaxone (2 g/day) for 7 days with a favorable outcome.

The strain was susceptible to penicillin G, rifampin, ciprofloxacin, and ceftriaxone, as determined by Etest method (bioMérieux, Florence, Italy). The breakpoints were those recommended by the Clinical and Laboratory Standards Institute (*7*). Serogroup was determined by serum agglutination, and serotype/subtype, NT:P1.15, 19 were determined by standard whole-cell ELISA with monoclonal antibodies (obtained from the National Institute for Biological Standards and Control, South Mimms, UK) (*8*).

PorA variable regions, FetA, and multilocus sequence typing analyses were performed according to standard procedures from the Neisseria Multi Locus Sequence Typing Web site (http://pubmlst.org/neisseria). The isolate from Italy had the pattern PorA VR1–19, VR2–15, and VR3–36; F5–5 and sequence type (ST)-2888. The same ST was already described in Greece in 2002 but in a noninvasive strain (http://pubmlst.org/neisseria).

The pattern obtained by pulsed-field gel electrophoresis (*9*), using the rare-cutting enzyme *Nhe*I, (data not shown), was identical to patterns found among meningococci X strains isolated in United Kingdom and belonging to ST-750, clonal group X-II (*1*). In particular, ST-2888 resembles, except for *gdh* gene sequence, ST-2317, which was found among the X meningococci isolated in the United Kingdom in 2002 with phenotype X:4:P1.7 (http://pubmlst.org/neisseria).

Our data document a rare case of invasive meningococcal meningitis in Italy, caused by *N. meningitidis* serogroup X ST-2888. Future surveillance data may be able to determine epidemiologic influences, likely emanating from nearby countries, on the spread of such a strain into Italy.
